# Virologic re-suppression and the associated factors among children aged 1-9 years on Antiretroviral Therapy in The Aids Support Organization Soroti Region, Uganda: a retrospective cohort analysis

**DOI:** 10.4314/ahs.v24i2.2

**Published:** 2024-06

**Authors:** Winfred Ruth Acham, Aisha Nalugya, Ricky Nyatia, Nelson Bunani

**Affiliations:** 1 Makerere University School of Public Health, Uganda; 2 The Aids Support Organization, Soroti, Uganda

**Keywords:** Virologic re-suppression, Human Immunodeficiency Virus, children

## Abstract

**Background:**

Children living with HIV experience low viral load re-suppression after a high viral load compared to the rest of the population. We determined the proportion with re-suppressed viral load and associated factors among children 1-9 years on Antiretroviral Therapy (ART) in The Aids Support Organization (TASO) Soroti Region.

**Methods:**

We conducted a retrospective cohort analysis of 401 records of children that initially had high viral load > 1000copies/ml for the period January 2016 to December 2018. Modified Poisson regression was performed to determine factors associated with virologic re-suppression.

**Results:**

The prevalence of virologic re-suppression was 97/401 (24.2%). More than half, 213 (53.1%) of the children were females and 197/401 (49%) were aged between 8 and 9 years. Factors associated with virologic re-suppression were; being on protease inhibitor (PI) based regimen [APR 2.87, 95% CI 1.76-4.79], good adherence [APR1.71, 95% CI 1.22-2.51] and caregiver HIV seropositive status [APR 2.56, 95% CI 1.69-3.91].

**Conclusion:**

Virologic re-suppression was low compared to the UNAIDS target. Taking PI-based regimen, good adherence and HIV seropositive status of the caregiver were predictors of virologic re-suppression. Close viral load monitoring of children on ART and intensified targeted adherence support to caregivers is vital to improving virologic re-suppression.

## Background

Globally, an estimated 1.8 million children aged 0-14 years were living with Human Immunodeficiency Virus (HIV) by the end of 2020, and of these, 53% were on antiretroviral therapy (ART)^1^. Sub-Saharan Africa (SSA) accounts for about 84% of all HIV infections among children globally^2^. In Uganda, 95,000 children were living with HIV in 2018, of whom 61,000 were receiving ART^3^,^4^. The Uganda Ministry of Health (MOH) adopted the WHO recommendations of routine Viral load (VL) monitoring for all patients on ART and recommended VL testing at six months of ART initiation and thereafter, every six months among HIV-positive children^3^,^5^. Despite routine VL monitoring, children in Uganda continue to experience the lowest VL suppression compared to the rest of the population^6^. The most recent population-based HIV impact assessment report found that VL suppression among children aged 0-14 years was 39.4 % compared to 59.6% among adults living with HIV^3^.

There have been pediatric ART program services in Uganda which include support for the children to stay adherent to medication through intensified adherence counselling (IAC) to their parents and caretakers^7^. Despite this, virological failure continues to manifest among children on ART even after their caretakers have received IAC. A recent study in Uganda found that only 23% of children (0-19 years) that completed IAC achieved viral load re-suppression^6^.

While the factors associated with virologic failure among the general population have been studied elsewhere^8^,^9^, the same can't be said for the children aged 1-9 years in Uganda, yet this is a vulnerable population that needs to be given attention. There is limited data on the number of children that get a re-suppressed viral load after virologic failure and the associated factors. We aimed to determine the proportion with a re-suppressed viral load and associated factors among children 1-9 years in TASO Soroti region, Uganda.

## Materials and methods

### Study design and area

This was a retrospective cohort study in which we quantitatively analyzed data of children aged 1-9 years receiving HIV care in Soroti region which is supported by TASO Uganda. TASO Uganda was established in 1987 to fight the HIV epidemic and provide free HIV services and free education to HIV-positive people. It currently has 11 centres in Uganda and supports the implementation of District Led programming to accelerate HIV prevention, care and treatment, for HIV/AIDS epidemic control in Soroti region. TASO Soroti region serves 10 districts in Northeastern Uganda where the prevalence of HIV is high at 4.2%^10^, yet the prevalence of viral load suppression is low compared to other areas in Uganda3. When disaggregated according to age, children have the lowest re-suppression compared to adolescents and adults^3^.

### Study population

We included HIV positive children aged 1-9-years on ART in TASO Soroti region active in care for at least six months or more from January 2016 to December 2018 and their caregivers. Children who had achieved virological suppression but whose viral load levels were elevated thereafter despite the availability of antiretroviral therapy were included in this study. We excluded children 1-9 years on ART with missing or inconclusive VL results.

### Sample size and selection of study participants

**Figure 1:**
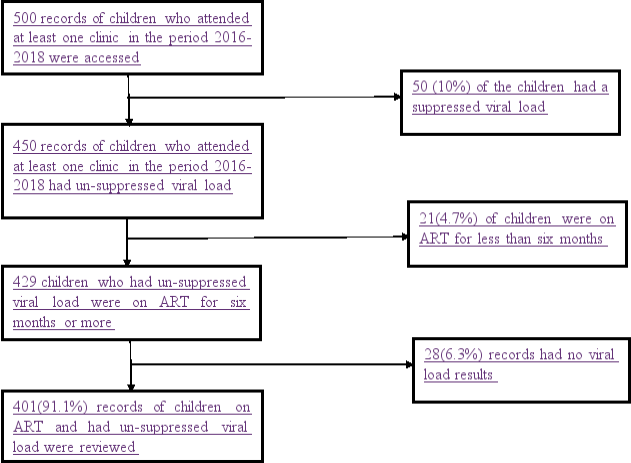
Flow chart for selection of children on ART

AA total of 500 records of children aged 1-9 years who attended at least one clinic in the period 2016-2018 were selected from 30 high volume health facilities out of 100 that provided HIV care in TASO-Soroti Region. Purposive sampling method was used in the selection of records for inclusion in this study. Ten per cent of the records were dropped because they indicated children had a suppressed viral load six months before the study. Four hundred fifty records indicated the children had an un-suppressed viral load, however, 21(4.7%) of these records were dropped because they indicated that the children had been on ART for less than six months. A total of 429 records of children who had been on ART for at least six months or more remained of which 28(6.3%) were dropped because they had no viral load results. Finally, a total of 401 records that met the inclusion criteria were considered for data abstraction. The selection criteria of the records of children are shown in figure 1.

### Study variables

The dependent variable was virologic re-suppression among HIV positive children 1-9 years in TASO Soroti region. This was determined based on any VL testing done with in four to six months period after adherence support following an initial non-suppressed VL as recommended by the guidelines^11^, ^12^. The guidelines recommend adherence support for three consecutive months following an initial high VL greater or equal to 1000 copies/ml, and then test again in the fourth month and up to six months to cater for delays in adherence support. The dependent variable was measured as a binary outcome where a viral load less than 1000copies/ml after six months of follow up was considered to be re-suppressed, while a viral load greater or equal to 1000copies/ml was considered not re-suppressed.

Independent variables included; sex, age of the child, age at initiation of regimen, ART regimen at initiation, duration on ART regimen, luster of Differentiation 4 (CD4), WHO stage, TB status and adherence, religion, education level, perceived confidentiality, stigma, support programs, occupation, caregiver HIV status and intensive adherence counselling (IAC). Adherence to ART was measured as that adherence recorded on the day of the most recent VL test, this was done basing on MoH guidelines^14^. According to the MoH guidelines adherence is measured as the percentage of prescribed medicines taken in the last one month. A person who had 2 doses remaining at the end of the appointment period was considered to have good adherence while that one with more than 2 doses was considered to have poor adherence.

The data were abstracted retrospectively from the patient records of children active in care from January 2016 to December 2018 using a data abstraction tool.

### Data management and analysis

The abstracted data were checked for completeness and then entered into Microsoft Excel 2016. Continuous numerical responses were entered as absolute values while categorical responses were coded. The coded data in Microsoft excel 2016 were exported to STATA version 15 for statistical analysis. This data was stored on a password-protected computer to ensure utmost confidentiality.

Data were analysed at univariate, bivariate and multivariable levels. Univariate analysis was done to describe the data. Categorical variables were summarized using frequencies and proportion, while continuous variables were summarized using the median and interquartile range. Modified Poisson regression with robust variances was then used at bivariate and multivariable analysis to identify factors associated with virologic re-suppression among children in TASO Soroti region. Associations were tested at 5% significance. A cut-off of p<0.2 was set to select variables at the bivariate analysis for the multivariable model based on the literature (15). All the variables that met the p<0.2 criteria at bivariate level were checked for collinearity, and added to the multivariable model using the stepwise elimination method to obtain adjusted prevalence ratios. Furthermore, factors that were above the cut off p<0.2 at bivariable model but were significant according to the literature were also considered for the multivariable model. A model with smaller Akaike's information criterion (AIC) and the log likelihood ratio closer to zero was considered to be the good fit. Unadjusted and adjusted prevalence ratios with their 95% confidence intervals were presented in bivariate and multivariable model tables.

## Results

### Socio-demographic and clinical characteristics

More than half, 213/401 (53.1%) of the children were females and 197/401 (49%) were aged between 8 and 9 years. The median age of the children was 8 (IQR 7-8) years. Furthermore, close to two-thirds, 251/401(62.6%) of the children were initiated on ART when they were aged 1-4 years and they mostly received NVP based regimen. Forty-one per cent of the children (n=166) had no known CD4 cell count while nearly half (48%, n=189/401) had WHO disease stage II. Majority, 268/401 (70%) of the participants had good adherence and 372/401(92.8%) of the children had received IAC. Most of the children 375/401(93.5%) had no TB signs. More than three quarters, 85.9% of the caregivers were peasant farmers and nearly half, 173/401(43.1%) were HIV seropositive. Furthermore, majority of the children 320(83.1%) resided in rural areas (Table 1).

**Table 1:** Socio-demographic and clinical characteristics of the study participants (N=401)

Factor	Virologic Re-suppression		
	Yes N (%)	No N (%)	Total N=401 (%)
**Sex**			
Male	50 (26.6)	138 (73.4)	188 (46.9)
Female	54 (25.4)	159 (74.6)	213 (53.1)
**Age**			
2-4	21 (31.8)	45 (68.2)	66 (16.5)
5-7	35 (25.4)	103 (74.6)	138 (34.4)
8-9	48 (24.5)	149(75.6)	197 (49.1)
**Age at initiation of ART**			
<1	25 (21.2)	93 (78.8)	118 (29.4)
1-4	72 (28.7)	179 (71.3)	251 (62.6)
5-9	7 (21.9)	25 (78.1)	32 (8.0)
**Current** ART regimen **			
EFV based	16 (16.5)	81 (83.5)	97 (24.3)
NVP	38 (20.5)	147 (79.5)	185 (46.4)
PI based	48 (41.0)	69 (59.0)	117 (29.3)
**CD4 count**			
CD4<250cells/ml	6 (20.0)	24 (80.0)	30 (7.5)
CD4 250<350 cells/ml	3 (16.7)	15 (83.3)	18 (4.5)
CD4350<500cells/ml	32 (47.1)	36 (52.9)	68 (16.9)
CD4≥500cells/ml	20 (16.8)	99 (83.2)	119 (29.7)
Unknown	43 (25.9)	123 (74.1)	166 (41.4)
**WHO stage**			
Stage I	44 (25.6)	128 (74.4)	172 (42.9)
Stage II	53 (28.0)	136 (72.0)	189 (47.1)
Stage III	2 (11.1)	16 (88.9)	18 (4.5)
Stage IV	5 (22.7)	17 (77.3)	22 (5.5)
Adherence**			
Poor	61(22.8)	207 (77.2)	268 (70.0)
Good	40 (34.8)	75 (65.2)	115 (30.0)
Occupation of the caregiver**			
Peasant	92 (28.5)	231(71.5)	323 (85.9)
Housewife	4 (23.5)	13 (76.5)	17 (4.5)
			
Teacher	2 (10.0)	18 (90.0)	20 (5.3)
Others*	2 (12.5)	14 (87.5)	16 (4.3)
**TB status**			
No signs or symptoms	98 (26.1)	277 (73.9)	375 (93.5)
TB suspect	4 (23.5)	13 (76.5)	17 (4.2)
Positive on TB treatment	2 (22.2)	7 (77.8)	9 (2.3)
**Caregivers HIV status**			
Unknown	32 (18.5)	141(81.5)	173 (43.1)
HIV seropositive	38 (31.2)	84 (65.9)	122 (30.4)
HIV seronegative	34 (32.1)	72 (67.9)	106 (26.4)
**IAC done**			
Yes	99 (26.6)	273(73.4)	372 (92.8)
No	5 (17.2)	24 (82.8)	29 (7.2)
Residence**			
Rural	82 (25.6)	238 (74.4)	320 (83.1)
Urban	17 (26.1)	48 (73.9)	65 (16.9)

* ***Other occupations included; tailors and market vendors***

** ***variables that had missing values***

Less than a third, 97/401 (24.2%) of the children had a re-suppressed viral load.

### Factors associated with virologic re-suppression

In bivariate analysis, the factors that were associated with virologic re-suppression were; being on PI based regimen [UPR 2.49, 95% CI 1.15-4.09], having a CD4 count (350<500cells/ml) [UPR 2.35, 95% CI 1.10-5.03], good adherence [UPR 1.53, 95% CI 1.09-2.13], and caregivers being HIV seropositive [UPR 1.21, 95% CI 1.10-2.54], and having an unknown HIV status [UPR 1.12, 95% CI 0.68-3.22].

In multivariable analyses, the factors that were associated with virologic re-suppression were; being on PI regimen [APR 2.87, 95% CI 1.76-4.79], good adherence [APR 1.71, 95% CI 1.22-2.51] and caregiver HIV seropositive status [APR 2.56, 95% CI 1.69-3.91]. (Table 2).

**Table 2:** Factors associated with virologic re-suppression among children 1-9 years on ART in TASO Soroti region

Factor	UPR (95%CI)	APR (95%CI)
**Sex**		
Male	1	
Female	0.95 (0.68-1.61)	
**Age**		
2-4	1	1
5-7	3.40 (0.88-13.21)	0.79 (0.46-1.54)
8-9	3.38 (0.88-13.03)	0.80(0.66-1.40)
**Age at initiation of ART**		
<1	1	1
1-4	1.35 (0.91-2.02)	1.12 (0.71-1.88)
5-9	1.03 (0.49-2.17)	0.088 (0.56-3.11)
**ART regimen at initiation**		
EFV based	1	1
NVP	1.25 (0.73-2.12)	1.34 (0.86-3.01)
PI based	2.49 (1.51-4.09)*	2.87 (1.76-4.79)**
**CD4 count**		
CD4<250cells/ml	1	
CD4 250<350 cells/ml	0.83(0.24-2.93)	
CD4350<500cells/ml	**2.35 (1.10-5.03)**	
CD4≥500cells/ml	0.84 (0.37-1.91)	
Unknown	1.30 (0.60-2.77)	
**WHO stage**		
Stage I	1	1
Stage II	1.10 (0.78-1.54)	0.86 (0.58-1.27)
Stage III	0.43 (0.11-1.65)	0.31 (0.11-1.35)
Stage IV	0.89 (0.39-2.00)	0.66 (0.22-2.11)
**Adherence**		
Poor	1	1
Good	1.53 (1.09-2.13)**	1.71 (1.22-2.51)*
**Occupation of the caregiver**		
Peasant	1	1
Housewife	0.83 (0.34-1.98)	1.13 (0.423.03)
Teacher	0.35 (0.93-1.32)	0.22 (0.12-1.70
Others	0.44 (0.12-1.63)	0.60 (0.12-3.10)
**TB status**		
No signs or symptoms	1	1
TB suspect	0.90 (0.38-2.16)	0.70 (0.520-3.01)
Positive on TB treatment	0.85 (0.25-2.93)	0.92 (0.33-4.01.)
**Caregivers HIV status**		
HIV seronegative	1	1
HIV seropositive	1.21 (1.10-2.54)*	2.56 (1.69-3.91)*
Unknown	1.12 (0.68-3.22)*	1.61 (0.16-2.98)
**IAC done**		
Yes	1	1
No	0.65 (0.29-1.47)	0.42 (0.30-1.33)
**Residence**		
Rural	1	
Urban	1.02 (0.65-1.60)	

UPR-Unadjusted Prevalence Ratio, * P< 0.05 ** p<0.001, IAC=Intensive Adherence Counseling, APR=Adjusted Prevalence Ratio CI=Confidence interval, EFV= efavirenz, NVP= nevirapine, PI= protease inhibitor.

## Discussion

The study aimed to determine the proportion of children that achieve a virologic re-suppression after a high viral load and the associated factors in TASO Soroti region. This study found that the proportion of children who re-suppressed following an initial high viral load in TASO Soroti was at 24.2% which was lower than the United Nations Program on HIV and AIDS (UNAIDS) target of 95%^16^. The current study registered a higher proportion of children with poor adherence which could be a contributor to low viral load re-suppression. The low viral load re-suppression may also be attributed to; missed clinic appointments which result in intermittent access to ART, drug-resistant mutations and adverse drug effects prompting caregivers to stop the medication^17^,^18^. Furthermore, children may not have re-suppressed because they depend on caretakers who may have not ensured that the children take their drugs. Comparable studies have also found that children and adolescents are significantly more likely to have a detectable viral load and less likely to re-suppress at retesting^19^. However, this re-suppression rate is lower than the proportion reported in Cambodia at 76.8% and South Africa at 70%^20^, ^21^. These countries have good viral load monitoring systems which could have led to viral suppression among the children. The higher proportion of viral re-suppression in Cambodia and south Africa is attributed to better healthcare infrastructure, greater access to ART, and more effective health education and awareness campaigns^1^,^2^.

Additionally, this study found that children who were taking the PI regimen had a high likelihood of achieving viral re-suppression. This is because the PI regimen is a superior drug to NVP/EFV and is therefore given to suppress the higher viral load^22^. Furthermore, the caretakers could have been anxious about their children being non-suppressed again, so they emphasize drug adherence more when the children have been switched to second line. The caretakers could have also been told by health workers that if children fail on second line, it would be harder to treat them as the third line treatment is difficult to get due to the associated costs^23^. Related findings were reported in South Africa where high proportions of viral suppression and medication adherence were found in this cohort of infants and young children initiating protease inhibitor-based antiretroviral treatment^21^. Another comparable study in the United Kingdom also found that the proportion of children who re-suppressed were those who were on PI-based first-line regimen because it has a high virus resistance barrier^24^.

The study further found that children who had good adherence to treatment had a high prevalence of achieving virologic re-suppression. Good adherence could have resulted in re-suppression because it maintains an optimal drug level and allows the drug to achieve the desired effects of suppressing viral replication thus promoting immune recovery. This is consistent with previous findings where poor adherence was reported to be associated with virologic failure^25^, ^26^. Another related study also found that poor adherence to HIV treatment was associated with drug resistance which affected viral re-suppression^27^.

Children whose caregivers were HIV positive were found to re-suppress after six months of follow up. This could have been due to the fact that these caregivers were also on treatment and therefore provide adherence support to the children. In addition, the caregivers who are HIV positive act as treatment reminders for the children^28^, ensure their timely VL bleeding, and also provide support for side effects since they could also be experiencing the same. These findings are consistent with findings from studies conducted in Harare and Myanmar where children that had been reported to have better viral outcomes were staying with a person living with HIV in the same household^29^, ^30^.

Although previous studies have found a significant association between virologic re-suppression and the age of the child, sex of the child, CD4 count, WHO disease stage, and IAC to the caregivers^18^, ^31^, this study did not find any association with these factors which may be attributed to contextual and design differences.^19^,^32^

## Strength and limitations

This study used secondary data, so human errors in data collection and entry may not be ruled out. We compared different data sources to correct these errors. Some of the information was missing in patient files. This posed a risk of having incomplete data. However, the number of files with missing information was small which did not affect our findings.

Purposive sampling was used to select the patient files which would have introduced a selection bias. This was minimized by correctly defining the target population, and reviewing all patient files that met the inclusion criteria.

While these results provide insight into the magnitude of the problem and possible factors in this population, they may not be fully generalizable to the general population and other settings.

## Conclusion and implications

The proportion of children that had a re-suppressed viral load after six months of follow up was lower than UNAIDS target. Being on PI-based regimen, having good adherence to treatment and HIV serostatus of the caregiver were predictors of virologic re-suppression.

## Data Availability

To protect the confidentiality of participant information, ethical restrictions have been imposed on the data used in this study. Interested researchers may submit queries related to data access to the HDREC at Mak-SPH (maksphhdrec@musph.ac.ug) or the corresponding author.

## List of abbreviations

IAC: Intensified Adherence Counselling
IRB: Institutional Review Board
HDREC: Higher Degrees Research and Ethics Committee
MOH: Ministry of Health
TASO: The Aids Support Organization
UBOS: Uganda Bureau of Statistics
UNAIDS: The Joint United Nations Program on HIV/AIDS
